# A neurotropic herpesvirus infecting the gastropod, abalone, shares ancestry with oyster herpesvirus and a herpesvirus associated with the amphioxus genome

**DOI:** 10.1186/1743-422X-7-308

**Published:** 2010-11-10

**Authors:** Keith W Savin, Benjamin G Cocks, Frank Wong, Tim Sawbridge, Noel Cogan, David Savage, Simone Warner

**Affiliations:** 1 Biosciences Research Division, Department of Primary Industries, 1 Park Drive, Bundoora, Victoria 3083, Australia; 2 Biosciences Research Division, Department of Primary Industries, 475 Mickleham Road, Attwood Victoria 3049, Australia; 3 Australian Animal Health Laboratory, CSIRO Livestock Industries, Geelong, VIC 3220, Australia; 4 School of Plant Biology, University of Western Australia, 35 Stirling Hwy Crawley, W.A 6009, Australia; 5 La Trobe University, Bundoora, Victoria 3086, Australia

## Abstract

**Background:**

With the exception of the oyster herpesvirus OsHV-1, all herpesviruses characterized thus far infect only vertebrates. Some cause neurological disease in their hosts, while others replicate or become latent in neurological tissues. Recently a new herpesvirus causing ganglioneuritis in abalone, a gastropod, was discovered. Molecular analysis of new herpesviruses, such as this one and others, still to be discovered in invertebrates, will provide insight into the evolution of herpesviruses.

**Results:**

We sequenced the genome of a neurotropic virus linked to a fatal ganglioneuritis devastating parts of a valuable wild abalone fishery in Australia. We show that the newly identified virus forms part of an ancient clade with its nearest relatives being a herpesvirus infecting bivalves (oyster) and, unexpectedly, one we identified, from published data, apparently integrated within the genome of amphioxus, an invertebrate chordate. Predicted protein sequences from the abalone virus genome have significant similarity to several herpesvirus proteins including the DNA packaging ATPase subunit of (putative) terminase and DNA polymerase. Conservation of amino acid sequences in the terminase across all herpesviruses and phylogenetic analysis using the DNA polymerase and terminase proteins demonstrate that the herpesviruses infecting the molluscs, oyster and abalone, are distantly related. The terminase and polymerase protein sequences from the putative amphioxus herpesvirus share more sequence similarity with those of the mollusc viruses than with sequences from any of the vertebrate herpesviruses analysed.

**Conclusions:**

A family of mollusc herpesviruses, *Malacoherpesviridae*, that was based on a single virus infecting oyster can now be further established by including a distantly related herpesvirus infecting abalone, which, like many vertebrate viruses is neurotropic. The genome of *Branchiostoma floridae *(amphioxus) provides evidence for the existence of a herpesvirus associated with this invertebrate chordate. The virus which likely infected amphioxus is, by molecular phylogenetic analysis, more closely related to the other 2 invertebrate viruses than to herpesviruses infecting vertebrates (ie chordates).

## Findings

In 2005 there was an outbreak of acute ganglioneuritis in an Australian population of the edible gastropod mollusc, abalone or *Haliotis *spp[1]. Using transmission electron microscopy, herpes-like particles were observed in ganglia of affected abalone[2] and purified virions from moribund abalone nervous tissues were identified as resembling those of herpesviruses, having an icosohedral capsid approximately 100 nm in diameter surrounded by a 150 nm diameter spiked envelope[3]. Potential herpesvirus particles were also identified previously in Taiwan following mortalities in *Haliotis diversicolor *[4]. Recently a diagnostic PCR test has been developed to detect the abalone virus [5]. The test has detected viral DNA sequences in diseased abalone from separate geographical locations in Australia and in DNA isolated from a herpes-like virus found some time ago in Taiwan[4].

Three herpesvirus families have been described in the order *Herpesvirales* - the *Herpesviridae *which infect *Mammalia*, *Aves *and *Reptilia*, the *Alloherpesviridae *infecting *Amphibia *and *Osteichthyes *(bony fish), and the mollusc-infecting *Malacoherpesviridae *containing a single virus that infects an invertebrate class, *Bivalvia *[6-8]. The phylogenetic relationships of these herpesviruses have been well studied and their evolution over epochs is largely synchronous with host lineages [7,8]. Gastropods separated early in the Cambrian period from all other known herpesvirus hosts. This unique evolutionary positioning[6] combined with our discovery of a related herpesvirus genome apparently integrated into the genome of another invertebrate, amphioxus, expands the *Herpesvirales *order and provides two key links to understanding the nature of the ancient ancestors of mollusc and vertebrate herpesviruses. To understand the structural and evolutionary relationships of the abalone virus to other herpesviruses, we purified abalone virus particles and isolated and sequenced genomic DNA using methods previously described[3,9]. The DNA was subjected to multiple displacement amplification[10] and sequenced using the Roche 454 GS-FLX system followed by partial genome assembly using the Newbler algorithm (Roche).

**Figure 1:**
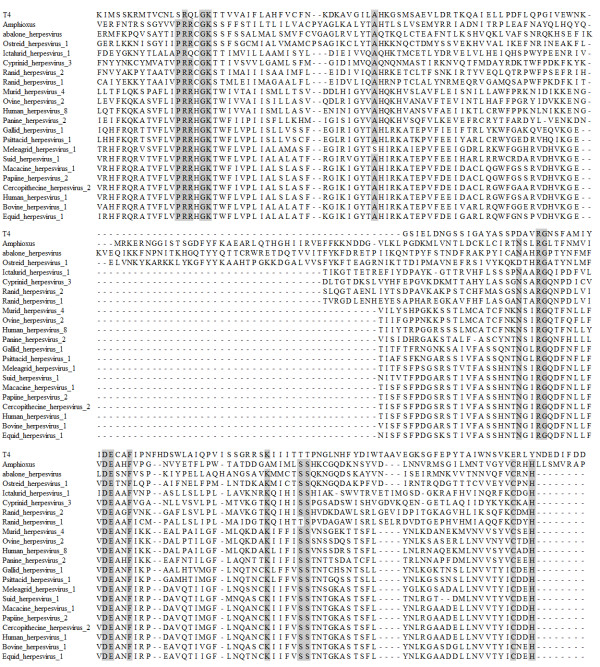
**Alignment of ATP hydrolase domains from terminase protein sequences**. ClustalW alignment of one of the conserved regions of the putative terminase gene - the ATP hydrolase (ATPase) domain from various herpesviruses taken from Table 3, identified using Interproscan. Grey background = >90% conserved amino acids.

**Figure 2:**
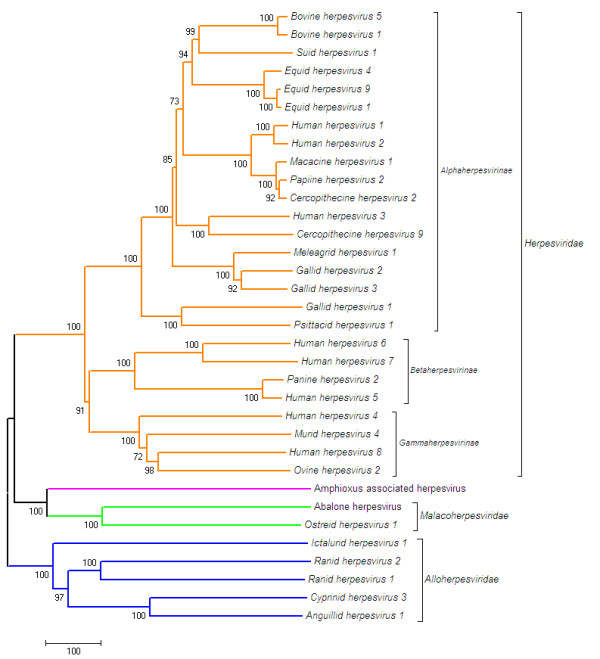
**Dendrogram of concatenated DNA polymerase and terminase protein sequences from 34 herpesviruses**. Dendrogram illustrating the evolutionary relationship of abalone and amphioxus herpesviruses to 32 other herpesviruses based on the concatenated full length protein sequences of DNA polymerase and the ATPase subunit of the putative terminase for each virus. The tree was inferred with MEGA4[32] using the Minimum Evolution (ME) method and a model based on the number of amino acid differences detected after an alignment using ClustalW[19]. The percentage of replicate trees in which the associated taxa clustered together in the bootstrap test (2000 replicates) are shown next to the branches. The scale bar for the branch lengths = 100 amino acid sequence differences.

Based on the assembled DNA sequences of the abalone virus, several protein coding sequences predicted using Artemis[11] showed varying distant homology to herpesvirus proteins, most notably those of *Ostreid herpesvirus *1 (oyster herpesvirus 1, OsHV-1), a virus infecting bivalve mollusc species[12,13]. BLAST analysis[14] of assembled sequence contigs based on predicted proteins identified 39 full length homologues of OsHV-1 genes (Table 1). These coding sequences, within partial genome scaffold sequences, or as individual coding sequences, have been submitted to Genbank. None of the coding sequences identified appear to be split by introns. Full-length sequences encoding homologues of DNA polymerase and the DNA packaging ATPase subunit of the (putative) terminase (henceforth referred to as the polymerase and terminase respectively), were identified and chosen for use in sequence alignments and phylogenetic analysis (Figures 1 &2). Hereafter, we will refer to the new abalone virus as abalone herpesvirus or AbHV-1.

**Table 1:** OsHV-1 homologues of AbHV-1 coding sequences

AbHV-1		OsHV-1		BLASTP result		
**Gene**	**Genbank**	**Genbank**	**Description**	**Ident.**	**Score**	**E value**

AbHVp002c	ADJ95315.1	YP_024647.1	ORF109 terminase	42%	620	3e-175

AbHVp003	ADL16651.1	YP_024565.1	ORF20 RNR2	37%	252	1e-64

AbHVp005c	ADL16652.1	YP_024602.1	ORF59	24%	90	1e-15

AbHVp006	ADL16653.1	YP_024573.1	ORF28	26%	239	2e-60

AbHVp013c	ADL16656.1	YP_024591.1	ORF47	27%	336	1e-89

AbHVp018c	ADL16657.1	YP_024590.1	ORF46	31%	48	6e-04

AbHVp019c	ADL16658.1	YP_024552.1 YP_024552.1	ORF49, ORF7 primase/helicase	24%, 24%	94, 74	5e-17, 1e-10

AbHVp024	ADL16662.1	YP_024567.1	ORF22	23%	234	7e-59

AbHVp031c	ADL16665.1	YP_024606.1	ORF66	27%	375	2e-101

AbHVp032	ADL16666.1	YP_024607.1	ORF67	32%	247	3e-63

AbHVp034	ADL16667.1	YP_024575.1	ORF30	27%	53	3e-05

AbHVp037c	ADL16668.1	YP_024616.1	ORF77	23%	170	1e-39

AbHVp038c	ADL16669.1	YP_024587.1	ORF43	27%	70	1e-10

AbHVp039c	ADL16670.1	YP_024634.1	ORF95	27%	94	2e-17

AbHVp043c	ADL16671.1	YP_024611.1	ORF71	23%	108	1e-21

AbHVp045c	ADL16672.1	YP_024604.1	ORF61	29%	185	1e-44

# AbHVp050	ADL16674.1	YP_024593.1, YP_024552.1	ORF49, ORF7 primase/helicase	21% 20%	125, 90	4e-26, 1e-15

AbHVp057c	ADJ95314.1	YP_024639.1	ORF100 DNA polymerase	31%	673	0.0

AbHVp064	HQ400676	YP_024619.1	ORF80		38.5	0.29

AbHVp070c	HQ400677	YP_024651.1	ORF113	25%	105	8e-21

AbHVp073c	HQ400678	YP_024650.1	ORF112	26%	119	7e-25

AbHVp075	HQ400679	YP_024649.1	ORF111	32%	198	6e-49

AbHVp086	HQ400681	YP_024645.1	ORF107	26%	134	4e-29

AbHVp093	HQ400682	YP_024622.1	ORF83	19%	49	3e-04

AbHVp102	HQ400683	YP_024630.1	ORF91	30%	151	1e-34

AbHVp104c	HQ400684	YP_024584.1	ORF40	30%	238	2e-60

AbHVp110	HQ400685	YP_024595.1	ORF52	34%	68	4e-10

AbHVp111	HQ400686	YP_024596.1	ORF53	23%	50	9e-04

AbHVp112	HQ400687	YP_024597.1, YP_024608.1	ORF54, ORF68	43%	643	0.0

AbHVp113c	HQ400688	YP_024657.1	ORF115	32%	80	3e-13

AbHVp117c	HQ400689	YP_024635.1	ORF96	23%	53	4e-05

AbHVp121	HQ400690	YP_024633.1	ORF94	28%	114	2e-23

AbHVp130c	HQ400691	YP_024605.1	ORF64	36%	212	6e-53

AbHVp131	HQ400692	YP_024615.1	ORF76	29%	202	1e-59

AbHVp133	HQ400693	YP_024569.1	ORF24	23%	67	3e-09

AbHVp134c	HQ400694	YP_024608.1, YP_024597.1	ORF68, ORF54	53%	784	0.0

AbHVp135c	HQ400695	YP_024624.1	ORF85	26%	225	1e-56

AbHVp136c	HQ400696	YP_024588.1	ORF44	32%	134	1e-29

AbHVp137	HQ400697	YP_024609.1	ORF69	29%	172	7e-41

Note: OsHV ORF49 & ORF7 are members of a gene family comprising ORF49, ORF7 & ORF115 OsHV ORF54 & ORF68 comprise a gene family.

AbHV Genbank accessions beginning with "AD" can also be found in scaffold sequences [Genbank:HM631981, Genbank:HM631982].

During the search for homologues of predicted AbHV-1 proteins using BLAST we identified, in the non-redundant (nr) Genbank protein sequence database, *Branchiostoma floridae *(amphioxus) coding sequences with significant homology to some of those in AbHV-1. The genome of amphioxus has been recently sequenced[15] although final assembly of chromosomes is not yet complete. On further analysis of amphioxus coding sequences using BLASTP with the predicted protein sequences of the oyster herpesvirus OsHV-1 genome (Genbank NC_005881), we identified 19 herpesvirus gene homologues. Consistent with this being an integrated virus, we found that 18 of these genes are clustered within a 150 kb region of a single amphioxus scaffold BRAFLscaffold_217, including the herpesvirus specific terminase gene[16] and all but 4 of these genes do not contain introns. These virus coding sequences appear to be legitimately assembled within published genome sequence scaffolds and are therefore probably integrated within the amphioxus genome. Further experiments such as fluorescence *in situ *hybridisation of chromosomes would confirm this. The 19 coding sequences identified are listed in Table 2 along with their OsHV-1 homologues and BLAST scores. We utilised the amphioxus virus terminase and polymerase protein sequence homologues in our analyses.

**Table 2:** *Branchiostoma floridae *(amphioxus) homologues of OsHV-1 coding sequences

OsHV-1	*Branchiostoma floridae*		BLASTP result		
**Accession/ORF**	**Accession**	**Location**	**Ident.**	**Score**	**E value**

YP_024639.1 ORF100 DNA polymerase	XP_002591163.1 DNA polymerase	55013..60373 no introns	28%	379	e-102

YP_024567.1 ORF22	XP_002591166.1	67372..72375 no introns	24%	128	8e-27

YP_024552.1 YP_024593.1 ORF7, ORF49 family primase-helicase	XP_002591168.1	76325..79696 no introns	24%	122	3e-25

YP_024630.1 ORF91	XP_002591169.1	80230..85225 introns predicted	29%	114	2e-23

YP_024606.1 ORF66 AE_Prim_S_like primase	XP_002591170.1	86185..88995 no introns	24%	239	2e-60

YP_024573.1 ORF28	XP_002591172.1	94244..96667 no introns	22%	107	4e-21

YP_024641.1 ORF102	XP_002591174.1	99529..101919 no introns	20%	78	4e-12

YP_024645.1 ORF107	XP_002591175.1 contains PAT1 domain pfam09770	103007..105292 no introns	24%	65	2e-08

YP_024584.1 ORF40	XP_002591176.1	105441..107045 no introns	29%	180	4e-43

YP_024643.1 ORF104	XP_002591178.1	108452..110641 no introns	19%	101	4e-19

YP_024615.1 ORF76	XP_002591179.1	112401..114281 no introns	26%	71	4e-10

YP_024624.1 ORF85	XP_002591189.1	137878..148379 introns predicted	22%	70	1e-09

YP_024597.1 YP_024608.1 ORF54, ORF68 family membrane glycoprotein	XP_002591190.1 XP_002591197.1 (possible gene family)	148508..150751 174789..176912 no introns	30%	332	1e-88

YP_024591.1 ORF47	XP_002591194.1	163504..167571 no introns	23%	275	4e-71

YP_024647.1 ORF109 terminase	XP_002591195.1 terminase	168081..170354 no introns	31%	308	2e-81

YP_024650.1 ORF112	XP_002591198.1	177489..179961 introns predicted	23%	68	2e-09

YP_024609.1 ORF69	XP_002591200.1	187709..188944 no introns	25%	80	3e-13

YP_024600.1 ORF57	XP_002610653.1 chloride channel	BRAFLscaffold_25 2304811..2311488 introns predicted	30%	86	4e-15

Note: *B. floridae *OsHV homologue locations are all on scaffold BRAFLscaffold_217, except for OsHV ORF57. All OsHV and *B. floridae *predicted proteins listed are of unknown function unless stated otherwise. Four *B. floridae *genes are predicted to contain introns. Also 4 other *B. floridae *genes in the scaffold BRAFLscaffold_217 between 60 kb and 150 kb encode proteins similar to apoptosis regulators like IAP-3 often present in herpesvirus genomes (not listed and not detected using OsHV sequences).

The putative terminase, or DNA packaging ATPase, appears specific to herpesviruses and some bacteriophages, such as T4[16] and is thought to be an enzyme motor involved in packaging viral DNA into preformed capsids[17]. We used the ATPase motif from this protein to investigate the phylogeny of the herpesviruses.

The ATP hydrolase (ATPase) motif sequences from 20 of the 34 terminase proteins listed in Table 3, plus their T4 bacteriophage homologue and the amphioxus terminase homologue (XP_002591195.1, listed in Table 2), were identified using Interproscan[18] and aligned using ClustalW[19]. Figure 1 shows that 12 amino acids are conserved across all herpesvirus ATPase domain sequences, including those from the abalone, oyster and amphioxus virus genomes, indicating the placement of the abalone virus and putative amphioxus virus within the *Herpesvirales *order. A common ancestral origin for the mollusc and amphioxus viruses is confirmed by the absence of introns in the terminase gene and the presence of additional amino acid loops (Figure 1). Although being in the same clade (Figure 2), at a protein sequence level the mollusc viruses are only moderately related with 40% amino acid identity in this conserved viral protein, across their full length.

**Table 3:** Genbank Accessions of Herpesvirus Polymerase and Terminase protein sequences used for phylogenetic analysis

Virus	Polymerase	Terminase
Abalone_herpesvirus	ADJ95314.1	ADJ95315.1

Amphioxus_associated_virus	XP_002591163.1	XP_002591195.1

*Anguillid_herpesvirus_1*	YP_003358194.1	YP_003358149.1

*Bovine_herpesvirus_1*	NP_045328.1	NP_045342.1

*Bovine_herpesvirus_5*	NP_954917.1	NP_954931.1

*Cercopithecine_herpesvirus_2*	YP_164473.1	YP_164457.1

*Cercopithecine_herpesvirus_9*	NP_077443.1	NP_077457.1

*Cyprinid_herpesvirus_3*	YP_001096114.1	YP_001096069.1

*Equid_herpesvirus_1*	YP_053075.1	YP_053090.1

*Equid_herpesvirus_4*	NP_045247.1	NP_045262.1

*Equid_herpesvirus_9*	YP_002333511.1	YP_002333526.2

*Gallid_herpesvirus_1*	YP_182359.1	YP_182378.2

*Gallid_herpesvirus_2*	AAF66765.1	YP_001033943.1

*Gallid_herpesvirus_3*	NP_066862.1	NP_066845.1

*Human_herpesvirus_1*	NP_044632.1	NP_044616.1

*Human_herpesvirus_2*	P07918.1	NP_044484.1

*Human_herpesvirus_3*	NP_040151.1	NP_040165.1

*Human_herpesvirus_4*	YP_401712.1	YP_401690.1

*Human_herpesvirus_5*	P08546.2	P16732.1

*Human_herpesvirus_6*	NP_042931.1	NP_042953.2

*Human_herpesvirus_7*	P52342.1	YP_073802.1

*Human_herpesvirus_8*	AAC57086.1	YP_001129382.1

*Ictalurid_herpesvirus_1*	NP_041148.2	NP_041153.2

*Macacine_herpesvirus_1*	NP_851890.1	NP_851874.1

*Meleagrid_herpesvirus_1*	NP_073324.1	NP_073308.1

*Murid_herpesvirus_4*	NP_044849.1	NP_044866.2

*Ostreid_herpesvirus_1*	YP_024639.1	YP_024647.1

*Ovine_herpesvirus_2*	YP_438136.1	YP_438152.1

*Panine_herpesvirus_2*	NP_612698.1	NP_612722.1

*Papiine_herpesvirus_2*	YP_443877.1	YP_443861.1

*Psittacid_herpesvirus_1*	NP_944403.1	NP_944422.2

*Ranid_herpesvirus_1*	YP_656727.1	YP_656697.1

*Ranid_herpesvirus_2*	YP_656618.1	YP_656576.1

*Suid_herpesvirus_1*	YP_068333.1	YP_068358.1

The phylogenetic analysis comparing concatenated polymerase and terminase full-length proteins (Figure 2, Table 3), illustrates the evolutionary relationships within the *Herpesvirales *order. The five *Alloherpesviridae *viruses are clustered together, with separate clades for frog and fish viruses as found previously [8], and the *Herpesviridae *are clustered into separate major clades reflecting their taxonomic groupings of alpha-, beta- and gammaherpesvirinae sub-families. The phylogenetic analysis confirms a relationship between the amphioxus virus and the abalone and oyster viruses in a deep invertebrate clade. The level of divergence makes estimation of the relative divergence times of the 3 herpesvirus families difficult. Interestingly, the amphioxus virus is in the clade with mollusc viruses, which may not have been expected given the amphioxus chordate host lineage is more aligned with vertebrates than molluscs.

The invertebrate herpesvirus clade provides a unique branching point to inform the metazoan diversification of the herpesviruses. It is thought that during the Cambrian era, the Bilaterial species diverged to generate the Protostomes (evolving into such animals as flatworms, molluscs and arthropods) and the Deuterostomes (from which the chordates and then the vertebrates evolved)[20,21]. Molluscs emerged more than 100 My before vertebrates with a bony skeleton (the current known range of herpesviruses in vertebrates). One hypothesis to explain the diversity of viruses within vertebrates and the positioning of the mollusc viruses among them, rather than as an ancestral outgroup, is the existence of diverse herpesviruses in Cambrian metazoans. Consistent with this hypothesis, previous estimates for the divergence of just the *Herpesviridae *in vertebrates indicate a divergence of alpha-, beta- and gammaherpesviruses to over 400 Mya, and longer times are predicted for divergence of *Alloherpesviridae *and *Malacoherpesviridae*[7]. An alternate hypothesis to explain the branching of the 3 herpesvirus families is that molluscs acquired herpesviruses by transmission in the aquatic environment, for example through association such as mollusc predation of early chordates. It appears that modern *Malacoherpesviridae *may have the ability to infect across species, a feature not typically observed in vertebrate herpesviruses, although the infection observed is restricted to related mollusc species[22].

As more sequence data and gene structure for *Alloherpesviridae*, *Malacoherpesviridae *and other invertebrate herpesviruses become available it will allow a more informative analysis of their evolution. Of particular interest will be new herpesviruses yet to be discovered in species which share bilateral symmetry such as amphioxus, sea squirts, flatworms or squid. Our discovery of clustered intact herpesvirus genes in amphioxus suggests an opportunistic integration has occurred in the amphioxus genome. This may not be a normal feature of infection and latency, but herpesviruses can occasionally integrate into the genome of their host[23]. Surprisingly, the nearest relatives of this chordate virus seem to be the viruses infecting molluscs rather than those of fish or frogs. Although herpesvirus particles have not been seen in the more primitive metazoan species, their existence is suspected; short herpes-like DNA sequences having been found in a metagenomic study of Hawaian coral[24]. Further metagenomic approaches similar to those described previously[25] and PCR-directed approaches[26] based on new sequences described here will enable these evolutionary questions to be addressed. The sequence information is also crucial for the development of molecular diagnostic tools to monitor and manage disease outbreaks.

The neurotropism of certain herpesviruses is well documented but this behaviour is not known outside the families of herpesviruses infecting terrestrial vertebrates[27,28]. The neurotropic tissue infection profile of the new gastropod virus analysed here is shared with some viruses within the *Herpesviridae *family. Convergent evolution may have given rise to the neurotropism seen in some members of the *Herpesviridae *and now the *Malacoherpesviridae *families. The rooting of a neurotropic invertebrate virus near or before the divergence of alpha-, beta-, and gammaherpesviruses, may also suggest that early mammalian herpesvirus precursors were neurotropic and that some have retained this feature over time. It is interesting to speculate as to the earliest functional interactions between sensory cells and viruses, as the first sign of neurons appeared over 600 million years ago in "cnidarians," (eg: hydra), but organisms basal to them like sponges do not have neurons or synapses[29]. Recent evidence indicates sponges have gene networks in cells which were precursors to nerve cells including proteins related to virus nerve entry receptors[30]. Others[24] have speculated on a link between herpesvirus neurotropism and the evolution of modern herpesviruses from ancestors infecting invertebrates such as *Cnidaria *(for example, coral or sea anemones), thought to be related to the first species with sensory receptors[31]. Further, the discovery reported here of a putative herpesvirus integrated into the genome of amphioxus hints at a wide diversity of herpesviruses within the invertebrate community, perhaps dating back to before the divergence of arthropods, molluscs and chordates. It will be exciting to discover such invertebrate herpesviruses and explore their links to ancient herpesvirus ancestors.

To accommodate the new abalone virus, which we have suggested naming abalone herpesvirus or AbHV-1, within the *Herpesvirales *order, we suggest the creation of a new genus called *Haliotivirus *within the *Malacoherpesviridae *family and assignment of AbHV-1 as a species under *Haliotivirus *(as *Haliotid herpesvirus 1*).

We have referred to the putative virus genome integrated into the *Branchiostomid *species chromosome as amphioxus-associated virus, AaHV-1. We suggest the species name *Branchiostomid herpesvirus 1*. Given the unique nature of the virus revealed by phylogenetic analysis and the unique evolutionary positioning of amphioxus as an invertebrate chordate, we suggest this virus, if classified, could be a member of a new family, Aspondyloherpesviridae (from the Greek for "no spine").

## Competing interests

The authors declare that they have no competing interests.

## Authors' contributions

KWS, FW, BGC, SW conceived and designed the experiments; FW, NC performed the experiments; KWS, TS, DS analyzed the data; FW, SW, TS, DS, NC contributed reagents, materials, analysis tools; KWS, BGC wrote the paper. All authors have contributed to the editing or revision of the manuscript and approve its publication.
